# Lymphoma B-cell responsiveness to CpG-DNA depends on the tumor microenvironment

**DOI:** 10.1186/1756-9966-32-18

**Published:** 2013-04-05

**Authors:** Rym Ben Abdelwahed, Jérémie Cosette, Sabrina Donnou, Lucile Crozet, Hanane Ouakrim, Wolf Herman Fridman, Catherine Sautès-Fridman, Aouni Mahjoub, Sylvain Fisson

**Affiliations:** 1 Institut National de la Santé et de la Recherche Médicale (INSERM), UMRS872, Centre de Recherche des Cordeliers, Paris F-75006, France; 2 Université Pierre et Marie Curie-Paris 6, UMRS 872, Paris F-75006, France; 3 Université Paris Descartes, UMRS 872, Paris F-75006, France; 4 Laboratoire MSC, Université Paris 7/CNRS, Paris, France; 5 Genethon, Evry, France; 6 INSERM, U951, Evry, France; 7 University of Evry Val d’Essonne, UMRS 951, 1 bis rue de l’Internationale, Evry, F-91002, France; 8 Faculté de Pharmacie de Monastir, LR99 ES27, Monastir, 5000, Tunisie; 9 Université de Monastir, Monastir, 5000, Tunisie

**Keywords:** TLR, CpG-DNA, Non-Hodgkin B-cell lymphoma, Subcutaneous lymphoma, Primary cerebral lymphoma, Primary intraocular lymphoma, Tumor microenvironment

## Abstract

**Background:**

Toll-like receptor (TLR) agonists have important properties that can be exploited for immunotherapy against tumors. Locally injected immunostimulatory oligodeoxynucleotides containing CpG motifs (CpG-ODNs), which are TLR9 agonists, have shown promise in cancer models. Several studies have demonstrated that these motifs have immunologic effects similar to those of bacterial DNA and can stimulate monocytes, macrophages, dendritic, and B cells, which then produce several proinflammatory cytokines. However, these CpG-ODNs appear to produce opposite effects on tumor B cells.

**Methods:**

In this study, we investigated the direct effects of a murine class B CpG (1826) ODNs on lymphoma B cells *in vitro* and *in vivo,* using mouse models of non-Hodgkin B lymphomas developing in immunoprivileged sites, specifically the brain and the eye, and in subcutaneous sites.

**Results:**

*In vitr*o, CpG-ODNs produced antiproliferative and proapoptotic effects on lymphoma B cells. *In vivo*, it had an antitumor effect when injected into tumors in murine models of subcutaneous lymphoma (SCL) and primary cerebral lymphoma (PCL). However, its intravitreal administration into a primary intraocular lymphoma (PIOL) mouse model did not produce an antitumor effect. *In vitro* experiments using supernatant from mouse PIOL samples demonstrated that the PIOL molecular microenvironment inhibits the antiproliferative effect of CpG-ODNs on lymphoma B-cells.

**Conclusions:**

Responsiveness to CpG stimulation differs in subcutaneous, cerebral, and ocular tumors, according to the tumoral and molecular microenvironment, and this should be considered for further therapeutic approaches.

## Background

Toll-like receptors (TLRs) are pattern recognition receptors that trigger innate and adaptive immune responses. Triggering TLRs activates a set of common proinflammatory genes and leads to the expression of antimicrobial effector cells and to production of inflammatory cytokines [1]. Agonists for TLRs have been identified and are being developed as new drugs and vaccine adjuvants to treat cancer, allergies, and infectious diseases [2]. In particular, oligodeoxynucleotides containing CpG motifs (CpG-ODN), which are TLR9 agonists, have shown promise against several types of tumors, including renal cell carcinoma, glioblastoma, melanoma, cutaneous T-cell lymphoma, and non-Hodgkin lymphoma [3]. Unmethylated CpG-DNA motifs have immunologic effects similar to those of bacterial DNA and can stimulate monocytes, macrophages, and dendritic and B cells; these then produce several Th1-type cytokines [4].

At least 3 structurally distinct classes of synthetic CpG-ODNs have been described, all capable of stimulating cells that express TLR9 [5,6]. CpG-B (also known as class-B CpG or K-type CpG) ODNs encode multiple CpG motifs on a phosphorothioate backbone and trigger the differentiation of antigen-presenting cells and the proliferation and activation of B cells [3]. Although the CpG-B motif is an established immunostimulatory agent, its direct effect on normal and tumor B cells seems to differ: CpG-ODNs stimulate proliferation of healthy B cells, activate their production of polyreactive immunoglobulins, and protect them from apoptosis [6-8]. On the other hand, these ODNs predominantly activate malignant B cells and then increase the rate of cell death, thus reducing survival of malignant B cells over time [9-11].

Different types of non-Hodgkin B-cell lymphomas differ in their responsiveness to CpG-DNA, and only limited information is available [9] about the sensitivity of malignant B cells to this DNA motif according to their *in vivo* microenvironment, particularly in immune sanctuaries such as the brain and eyes. Unlike systemic lymphoma, primary cerebral lymphoma (PCL) and primary intraocular lymphoma (PIOL) are subsets of primary central nervous system lymphoma (PCNSL), and they affect immunologically privileged organs. Both usually appear as a diffuse large B-cell non-Hodgkin lymphoma in which malignant lymphoid cell types not normally present in the brain or eye are detected [12]. The internal tissues of the brain and eye are usually protected from the inflammatory processes mediated by the immune system.

In this study, we compare the effect of CpG-ODNs on cerebral and ocular diffuse large B-cell lymphoma and on subcutaneous lymphomas (SCL). We show that A20.IIA murine B-cell lymphoma expressed high levels of endogenous TLR9 protein that produced an antiproliferative effect when stimulated *in vitro* by CpG-ODNs. A proapoptotic effect accompanied this reduced proliferation. *In vivo* local administration had a similar antitumor effect on subcutaneous and cerebral lymphomas. However, local administration of CpG-ODNs in a PIOL mouse model did not produce an antitumor effect. *In vitro* experiments with supernatant from ocular lymphoma samples demonstrated that the molecular microenvironment of PIOL counteracts the direct antiproliferative effect of CpG-ODNs on lymphoma B-cells. These findings show that cerebral and ocular tumor cells differ in their responsiveness to CpG stimulation according to the tumor environment. The microenvironment of the eye must be further characterized to identify the negative regulators.

## Methods

### Reagents

Nuclease-stable phosphorothioate-modified CpG 1826 (CpG) with 5_-TCCATGA**CG**TTCCTGA**CG**TT (the nucleotides in bold represent the immunostimulatory CpG sequences), fluorescein isothiocyanate (FITC)-conjugated CpG 1826 ODNs, and control 1826 ODN with 5_-TCCATGAGCTTCCTGAGCTT were purchased from InvivoGen (Cayla, France).

### Cells

A20.IIA is an FcγR-negative clone originating from the A20-2 J B-cell lymphoma line [13]. For *in vivo* experiments, A20.IIA cells were transfected by an electroporation system with the green fluorescent protein (GFP) gene. These cells, hereafter referred to as A20.IIA or A20.IIA-GFP cells as appropriate, were maintained at 37°C, 5% CO_2_ in complete Roswell Park Memorial Institute (RPMI) 1640 Medium Glutamax plus (RPMI; Gibco-Invitrogen, France) supplemented with 10% fetal calf serum (FCS; PAA Laboratories, Germany), 100 μg/mL penicillin and 100 μg/mL streptomycin (both from Eurobio, France), 10 mM sodium pyruvate (Gibco-Invitrogen), and 50 μM 2-mercaptoethanol (Gibco-Invitrogen). The A20.IIA-GFP cell culture was also supplemented with 0.5 mg/mL neomycin (G418; Gibco-Invitrogen). To obtain the A20.IIA-*luc*2 cell line, A20.IIA cells were transfected with pGL4.50[luc2/CMV/hygro] (Promega), in the AMAXA Nucleofector II device (Lonza, Switzerland) and were cultured in 0.75 mg/mL hygromycin B (Gibco-Invitrogen) medium.

### Proliferation assay

A20.IIA cells at a concentration of 10^5^cells/mL were incubated with serial dilutions of CpG 1826 or control 1826 ODNs at concentrations ranging from 0.0003 to 60 μg/mL or with complete RPMI medium alone. After 3 days, [^3^H] thymidine (GE Healthcare) was added for the last 4 h. Cells were harvested onto fiber filters and [^3^H] thymidine incorporation was measured in a scintillation counter (Microbeta, Perkin Elmer).

### Apoptosis assay

A20.IIA cells (10^4^) were cultured in complete RPMI medium in 96-well plates in the presence or absence of 3 μg/mL or 30 μg/mL of CpG or control ODNs. Staining with Annexin V/allophycocyanin (APC) and propidium iodide (PI) (BD Biosciences, France) was performed 72 h later and then analyzed by flow cytometry. Apoptotic cells were defined as those positive for Annexin V and PI.

### Mice

Female BALB/c mice (H-2^d^) were obtained from Charles River Laboratories (L’Arbresle, France) and used between 6 and 8 weeks of age. They were provided with sterile food and water *ad libitum* and kept on a 12-hour light–dark cycle. All procedures involving mice conformed with European Union guidelines, French regulations for animal experimentation (Ministry of Agriculture Act No. 2001–464, May 2001), and the guidelines of the Institut National de la Santé et de la Recherche Médicale Committee on Animal Research, and were approved by the relevant local committees (Charles Darwin Ethics Committee for Animal Experiments, Paris, France; Permit Number: p3/2009/004).

### Tumor implantation

Mice were first anesthetized by intraperitoneal injection of a mixture containing 120 mg/kg of ketamine (Virbac, France) and 6 mg/kg of xylazine (Rompun 2%; Bayer Healthcare). To obtain a subcutaneous lymphoma (SCL) murine model, BALB/c mice were inoculated subcutaneously with 5 × 10^6^ A20.IIA-GFP tumor cells in a final volume of 50 μL of RPMI, at 2 different sites: the right and left abdomen. For the intracerebral tumor implantation, anesthetized mice were immobilized on a stereotaxic frame (David Kopf Instruments, Tujunga, CA, USA). Tumor cells (5 × 10^4^ in a final volume of 2 μL RPMI) were injected into the specific cerebral location (right striatum), located 2 mm to the right of the medial suture and 0.4 mm in front of the bregma, through a Hamilton syringe attached to a penetrating depth controller. The penetrating depth of the syringe was 2.5 mm from the surface of the brain. Each injection delivered the solution slowly, and the syringe was held in place for an additional minute to reduce backfilling of tumor cells. For the intravitreal tumor implantation, we used a 32-gauge needle attached to a syringe to inject 10^4^ cells in a final volume of 2 μL of RPMI into the vitreous under a dissecting microscope. Lacrinorm 2% (Bauch&Lomb) drops were instilled after intravitreal injection. For each tumor model, control mice received either 1× phosphate-buffered saline (pH7.4; PBS) or control 1826 ODNs instead of CpG 1826 ODNs.

### Treatment injections

Tumor growth in the SCL model was monitored by caliper measurements 3 times a week. Treatment began when the longest tumor diameter reached 0.5 to 0.7 cm. The mice then received daily intratumor injections of CpG-ODNs for 5 days (100 μg per injection in a final volume of 50 μL RPMI) in the right tumor only; the left tumor served as an untreated control tumor. Mice were killed one week after the last treatment injection. Lymphomas established in the brain and eye were treated 7 days after tumor inoculation, by a single local injection of 60 μg (brain) or 20 μg (eye) CpG-ODNs in 2 μL of RPMI (treatment groups) or 2 μL of PBS (control groups). Tumor burden was analyzed in the sacrificed mice one week after treatment administration.

### Isolation of brain, ocular and subcutaneous lymphomas

The tumor-injected brains and eyes and the subcutaneous tumors were harvested one week after treatment injection, minced with surgical scissors, incubated for 30 minutes in RPMI containing 0.1 mg/mL DNAse I (Roche Diagnostics, Meylan, France) and 1.67 Wünch U/mL Liberase (Roche), and filtered through a 70-μm membrane (BD Falcon). Mononuclear cells were separated from myelin with a Percoll cell density gradient.

### *In vivo* tumor growth assay

The A20.IIA (1 × 10^4^) cells expressing luciferase (luc2 gene) were injected via subcutaneous, intracerebral or intravitreal routes into immunocompetent 7-week-old BALB/c mice. CpG or control ODNs were administered in *situ* for each lymphoma model according to the same experimental design and at the time points and doses described above. The tumor burden was thereafter monitored by bioluminescence imaging. Mice were injected intraperitoneally with 150 mg/kg of D-luciferin potassium salt (Interchim) and underwent imaging within the next 10 minutes with the IVIS LUMINA II (Caliper LS) imaging system. The exposure time was set to optimize the signal and obtain the best signal-to-noise ratio. The bioluminescence signal is expressed in photons per second.

### Supernatant harvesting

Mice were implanted with tumor cells in the brain (PCL), eye (PIOL) or flank (SCL) or injected with PBS in the eye (PIE). Either 14 days later (brain and eye) or when tumor diameter reached 0.5 to 0.7 cm (SCL), the relevant cells were isolated and cultured for 36 h in complete RPMI medium in 96-well (10^4^ cells per well) plates. Supernatant was then harvested from each well.

### Flow cytometry

To analyze TLR9 expression on A20.IIA cells, these cells underwent intracellular staining with the Fixation/Permeabilization solution kit (BD Biosciences) and an anti-TLR9/PE mAb (BD Biosciences).

Tumor burden was analyzed according to the following protocol: Fc receptors were saturated for 20 min with 10 μg/mL of anti-CD16/CD32 mAb (clone 2.4.G2), and then the cells were incubated for 20 min with either rat IgG2a anti-CD19/APC mAb, or the corresponding isotypic mAb control (all from BD Biosciences). The living cells were defined with side scatter (SSC) and forward scatter (FSC) after autofluorescent cells were excluded. Cell phenotypes were analyzed with the LSRII cytometer and Diva software (BD Biosciences).

### Statistical analysis

Comparisons used Student’s t-test, performed with GraphPad Prism (GraphPad Software, La Jolla, CA, USA). Statistical significance was defined by *p* values less than 0.05.

## Results

### CpG-ODNs inhibit cell proliferation and induce apoptosis of malignant A20.IIA B cells *in vitro*

**Figure 1:**
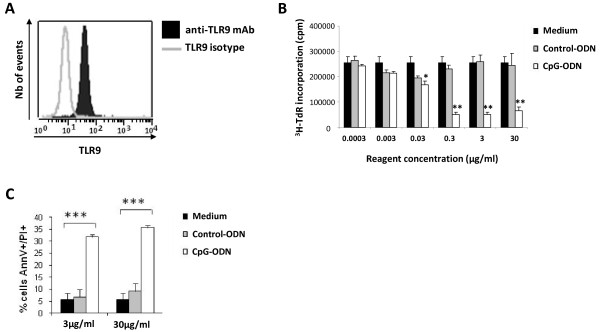
**CpG inhibits cell proliferation and induces apoptotic death of A20.IIA lymphoma cells *in vitro*.** (**A**) Flow cytometric analysis of TLR9 expression by A20.IIA cells after anti-TLR9 Ab staining (filled histogram), overlaid with isotype control (gray line). (**B**) CpG inhibits the proliferation of A20.IIA cells *in vitro*. 10^4^ A20.IIA cells were stimulated for 72 hours with various concentrations of CpG or control ODNs ranging from 0.0003 to 30μg/mL or with medium alone. The incorporation of the [^3^H] thymidine was measured by a scintillation counter. *P < 0.05; **P < 0.01. The data shown are representative of 1 of 3 experiments. (**C**) CpG induces apoptotic cell death of A20.IIA cell line. Cells were incubated for 72 hours with CpG or control ODNs at 3 and 30 μg/mL, or medium alone. The percentage of AnnV/PI positive cells was determined by flow cytometric analysis. ***P < 0.001.

TLR9 is an intracellular receptor that recognizes CpG-DNA. Cell stimulation by CpG motifs requires that they bind to TLR9. We therefore began by confirming with flow cytometry that A20.IIA B lymphoma cells express TLR9 (Figure 1A).

We next evaluated the direct effect of CpG-ODNs on the proliferation of A20.IIA lymphoma *in vitro*. Based on our study of its proliferation kinetics (data not shown), tumor cells were incubated for 72 h with CpG 1826 ODNs at concentrations ranging from 0.0003 to 30 μg/mL. Cell proliferation was measured with the [^3^H] thymidine incorporation assay. The CpG-ODNs inhibited A20.IIA [^3^H] thymidine incorporation in a dose-dependent manner, whereas control ODNs had no effect on cell proliferation (Figure 1B). The maximum inhibitory effect was obtained from 0.3 to 30 μg/mL of CpG-ODNs.

Based on these results, we analyzed the induction of apoptosis of A20.IIA cells by CpG- ODNs and found that they induced apoptosis of about 30 to 35% of the cells (that is, 30-35% stained positive for Annexin V and PI), compared with 5% for RMPI Medium and 5-10% with the control ODNs (Figure 1C).

### Lymphoma cell responsiveness to CpG sequences differs according to their tissue microenvironment

**Figure 2:**
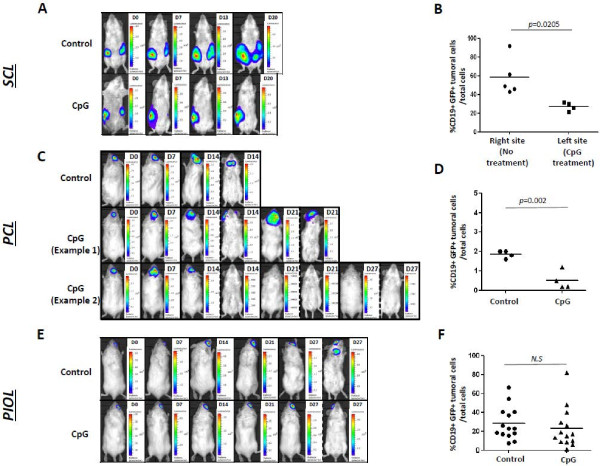
**CpG-ODNs decrease the burden of subcutaneous and cerebral tumors but fail to induce PIOL regression.** The 2-tumor-site SCL model: (**A**) Representative bioluminescence images of SCL treated with control ODNs (upper panel) and CpG-ODNs (lower panel). The mice were injected with 5x10^6^ A20.IIA-GFP-luc2 cells. Treatment was injected *in situ* when the tumor reached 0.5 to 0.7 cm in diameter. (**B**) Flow cytometric analysis of GFP^+^ CD19^+^ tumor cells, 7 days after the end of CpG-ODN administration in right tumors compared to left (untreated) tumors. PCL lymphoma model: (**C**) Representative bioluminescence images of PCL mice treated with control ODNs (upper panel) and CpG-ODNs (both lower panels), showing 2 different profiles of responsiveness to CpG motifs. The mice were injected with 5x10^4^ A20.IIA-GFP-luc2 cells and treated one week after tumor inoculation. (**D**) The percentage of CD19^+^ GFP^+^ tumor cells, as determined by flow cytometry, in the brain of mice treated with CpG at 60 μg/2μL, in comparison with PBS 1X (Control)-injected mice (n = 5 per group). PIOL lymphoma model: (**E**) Representative bioluminescence images of PIOL mice treated with control ODNs (upper panel) and CpG-ODNs (lower panel). The mice were injected with 10^4^ A20.IIA-GFP-luc2 cells. CpG-ODN treatment was administered on day 0 intravitreously. (**F**) Flow cytometric analysis of the percentage of GFP^+^CD19^+^ tumor cells in PIOL-inoculated right eyes (n = 14 per group). A week after tumor injection, right eyes of mice were treated intravitreally by 20 μg/2 μL of mice injected with CpG-ODNs or PBS 1X (Control). Results obtained from 2 independent experiments were pooled. Statistical test: Mann–Whitney; NS: not significant.

After showing that the CpG motif has a direct antiproliferative and proapoptotic effect on A20.IIA lymphoma cells, we sought to explore its effects *in vivo* when injected intratumorally, by comparing the 3 types of murine models of lymphoma: SCL, PCL and PIOL. A20.IIA-GFP cells were implanted on the left and right flank of the mice for the SCL model. Tumor size was measured by a caliper 3 times a week. When the tumors reached 5–7 mm in diameter, the left site was treated by local injections of CpG- ODNs, while the right one was used as an untreated control tumor. As described by Houot & Levy in 2009 [14] mice did or did not receive daily intratumoral injections of 100 μg/50μL CpG-ODNs for 5 days. Tumor size was then measured daily until sacrifice, one week after the last treatment injection. The tumor burden of mice treated with CpG and control ODNs was compared with a bioluminescence imaging system that assessed total photon influx. The CpG-ODNs inhibited tumor growth very soon after treatment in this SCL model. On day 7 after treatment, the untreated tumor was more than 100 times brighter than the CpG-treated one, and on day 20, 120 times brighter (Figure 2A). Flow cytometric analysis of CD19^+^GFP^+^ cells confirmed that tumor cells decreased significantly more in the treated than the untreated tumors (Figure 2B).

We next addressed the question of whether CpG motifs have the same antitumor effect in cerebral lymphomas. Imaging analysis showed two different profiles. Some mice did not respond to *in situ* CpG-ODN treatment, and the lymphoma developed in the brain and even developed in lymph nodes at day 21; this timing was nonetheless later than in the control group (Figure 2C – Example 1). Some mice did respond to the treatment; the tumor grew from day 0 to day 7 after treatment, and then decreased until it was undetectable (Figure 2C – Example 2). We also examined the percentage of CD19^+^GFP^+^ cells in the group treated by CpG-ODNs, compared it with the control group and observed a significant decrease in the proportion of tumor cells (Figure 2D).

Next we investigated the antitumor effect of CpG-ODNs on PIOL mice that had a tumor implanted in the right eye only and were then treated with CpG-ODNs (20 μg/2μL) or control ODNs (20 μg/μL). As shown in Figure 2E, CpG-ODNs seem to have had no detectable effects on the primary eye tumor. Nevertheless, they appeared to prevent lymph node invasion at day 27 (Figure 2E). Flow cytometric analysis showed no significant difference in tumor growth between CpG ODN-treated and control (PBS 1X) treated eyes (Figure 2F).

These results suggest that the behavior of tumors in the eye is different from that of systemic lymphomas, but also from that of cerebral lymphoma, and thus, that tumor cells responsiveness to CpG-DNA depend on the tissue microenvironment.

### Soluble molecules from the PIOL microenvironment counteract the antiproliferative effect of CpG-ODNs on malignant B-cells in a dose-dependent-manner

**Figure 3:**
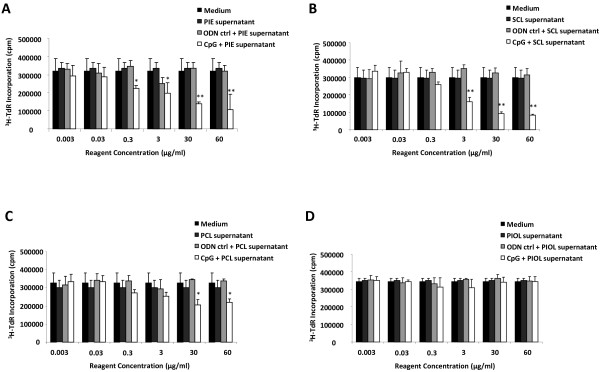
**PIOL supernatant counteracts *in vitro *antiproliferative effect of CpG-ODNs on A20.IIA malignant B cells.** 10^4^ cells were stimulated for 72 hours with various concentrations of CpG or control ODNs in concentrations ranging from 0.003 to 60 μg/mL or with medium alone and with the presence of supernatant from (**A**) PBS 1X injected eyes (PIE), (**B**) SCL, (**C**) PCL, or (**D**) PIOL. The incorporation of the [^3^H] thymidine was measured by a scintillation counter. *P < 0.05; **P < 0.01. The data shown are representative results from 1 of 3 experiments.

**Figure 4:**
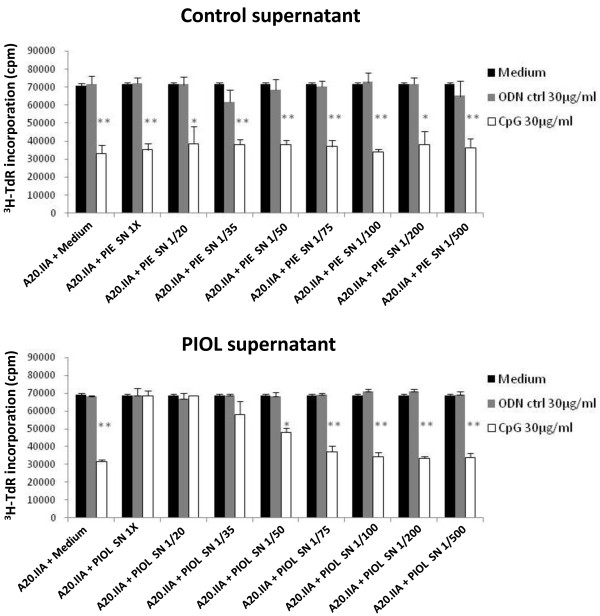
**Soluble molecule present in PIOL but not in normal ocular microenvironment is able to abrogate *in vitro *effect of CpG-ODNs in a dose-dependent manner.** 10^4^ cells were stimulated for 72 hours with CpG or control ODNs at 30 μg/mL and in the presence of several diluted doses of control supernatant (PIE) or PIOL supernatant (1X, 1/20, 1/35, 1/50, 1/75, 1/100, 1/200, 1/500). The incorporation of the [^3^H] thymidine was measured by a scintillation counter. *P < 0.05; **P < 0.01.

As described above, *in vivo* experiments showed that the responsiveness of lymphoma B cells to CpG-ODN administration was tissue-dependent. To confirm that the blockade of CpG-ODN antitumor effects was due to the PIOL molecular microenvironment, we tested whether supernatant from PIOL could counteract the inhibitory effect of CpG-ODNs on the proliferation of A20.IIA cells *in vitro*. A [^3^H] thymidine incorporation assay was performed as described above, with the addition of supernatant obtained from PBS-injected eyes (PIE) (as control), or from the mouse model SCL, PCL, and PIOL. As shown in Figure 3, the addition of PIE (Figure 3A) and SCL (Figure 3B) supernatants did not modify the ability of CpG-ODN treatment to inhibit tumor growth. PCL supernatant (Figure 3C) increased proliferation, but CpG-ODNs were still active at doses of 30 and 60 μg/mL. In contrast, CpG-ODNs were unable to inhibit tumor cell proliferation after incubation with PIOL supernatant (Figure 3D) and to induce apoptosis (data not shown). Moreover, their antiproliferative effect was recovered upon dilution of the PIOL supernatant (Figure 4). These data confirm our *in vivo* results and show that a soluble factor, released in eye tumors but not in normal eyes, was able to counteract the antiproliferative effect of CpG motifs.

### The PIOL microenvironment did not modify either TLR9 expression or the internalization of CpG-ODNs by tumor cells

**Figure 5:**
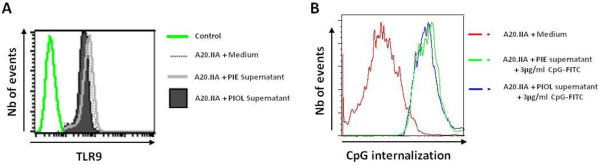
**The PIOL microenvironment did not modify TLR9 expression or internalization of CpG-ODNs by tumor cells.** (**A**) 10^4^ A20.IIA cells were incubated with PIOL or PIE supernatant. 3 days later, cytometric analysis was performed of TLR9 expression by cells incubated with PIOL supernatant, overlaid with isotype control and compared to TLR9 expression by cells incubated with PIE supernatant or medium alone. (**B**) 10^4^ A20.IIA cells were incubated for 24 hours with medium alone or with PIOL or PIE supernatant and in the presence or absence of FITC-labeled CpG-ODNs at 3 μg/mL. FITC expression by A20.IIA tumor cells was analyzed by flow cytometry.

To investigate the possibility that the loss of the CpG-ODNs antitumor action was associated with modulation of TLR9 expression, we used flow cytometry to compare TLR9 expression on A20.IIA cells after incubation with supernatant from medium alone, PIOL or PIE. No differences were found between these conditions (Figure 5A).

Next we examined whether the PIOL molecular microenvironment inhibited internalization of CpG ODNs by tumor cells. FITC-labelled CpG 1826 ODNs were added for 24 hours at a concentration of 3 μg/mL to A20.IIA lymphoma cells in the presence of PIOL or PIE supernatant. Flow cytometric analysis indicated that FITC expression by tumor cells with PIOL supernatant was similar to that incubated with PIE supernatant (Figure 5B).These findings show that the addition of PIOL supernatant does not modify CpG internalization by lymphoma B-cells, even *in vivo* in our three model (data not shown).

## Discussion

The ultimate goal of cancer immunotherapy is to eradicate tumors through vaccine strategies. Recent studies have demonstrated that synthetic CpG-ODNs induce regression of highly immunogenic tumors by engaging both the innate and the adaptive immune systems. CpG-ODNs are currently being tested in clinical trials for the treatment of non-Hodgkin B-cell lymphoma, which expresses TLR9 [15]. However, only limited information is currently available about the sensitivity to CpG-ODNs of primary malignant B-cells of different non-Hodgkin lymphoma entities. Understanding their direct effect on malignant B-cells is important as we consider how this potent class of agents might be used in the immunotherapy of lymphoma.

Here, we found that A20.IIA malignant murine cells, related to diffuse large B cells, express TLR9 and are sensitive to CpG-B ODN stimulation *in vitro*. As reported previously, CpG-ODNs induce a dose-dependent antiproliferative effect [16] and increase apoptotic cell death [17]. This apoptosis has been described as caspase-dependent and is accompanied by up-regulation of CD95/Fas and its ligand [9]. Another group demonstrated that TLR9 signaling by CpG-B ODNs leads to NF-kB-dependent production of autocrine IL-10, which then activates JAK/STAT pathway-dependent tyrosine phosphorylation of STAT1 proteins and thereby engenders an apoptotic pathway in human chronic lymphocytic leukemia B-cells [10]. Comparing primary B-cell lymphomas from patient samples, other authors have showed that cell responsiveness to CpG-ODNs varies, with different degrees of activation and apoptosis induction [9]. Several studies have reported that CpG-ODNs induce activation of normal B-cells and block apoptosis [7]. Although the molecular mechanisms of these effects remain unclear, it has been suggested that reactive oxygen species (ROS) and NFkB activation may play a role [18].

An important question is whether the *in vitro* responses to CpG motifs that have been observed could produce an *in vivo* antitumor effect on DLBCL lymphoma mouse models. We used 3 mouse models to begin to answer this question: a primary systemic lymphoma model (subcutaneous lymphoma) and 2 primary central nervous system lymphoma subtypes (cerebral and ocular lymphoma mouse models). The brain and eyes, considered to be immune sanctuaries, are relatively isolated from the systemic immune system by anatomic and physiologic barriers that maintain a local immune tolerance to protect neuronal cells from inflammation [19]. The use of these different models allowed us to compare the responsiveness to CpG-ODNs of the same tumor cells located in different immune microenvironments.

Thus, we demonstrated that local administration of CpG-ODNs into subcutaneous lymphoma decreased the tumor burden. This effect is probably attributable to immune cell activation of NK cells and DCs, which activates innate and adaptive immunity. In addition, the CpG-ODNs inhibited proliferation and induced apoptosis of TLR9-positive tumor cell lines *in vitro*. Interestingly, intratumor administration of CpG-ODNs induced tumor regression of cerebral but not intraocular lymphoma. The similarity in origin and classification of PCL and PIOL, which are related subsets sharing the particularity of developing in different immune-privileged sites, makes these results especially striking.

In addition, PIOL supernatant selectively abrogated the inhibitory effects of CpG-ODNs *in vitro,* in contrast to supernatant from nonmalignant eyes (PBS-injected eye) or SCL. PCL supernatant, on the other hand, had an intermediate inhibitory effect on the *in vitro* antiproliferative action of CpG-ODNs. Together, these data suggest that soluble factors are produced in the PIOL microenvironment, to a lesser degree in the PCL microenvironment, and not at all in subcutaneous microenvironment. These factors can inhibit the effect of this TLR9 agonist on lymphoma B-cells. This inhibition was not due to downregulation of TLR9 expression or to a blockade of CpG internalization by tumor cells. Further investigation is needed to characterize TLR9-mediated signaling and molecular mechanisms that might differ in the PIOL microenvironment.

## Conclusions

In conclusion, we showed here that, in addition to their immune-enhancing effects, CpG-ODNs inhibit lymphoma B cell proliferation and induce apoptotic cell death *in vitro*. They also reduced tumor growth in systemic and cerebral lymphomas *in vivo*. These findings support the value of developing TLR9-targeted therapy with CpG-B ODNs as a therapeutic agent for primary non-Hodgkin B-cell lymphoma. Further investigation should seek to identify and characterize the soluble factors from the PIOL microenvironment that inhibit the effects of CpG-ODNs and enable us to understand the potential immunosuppressive effect on host immune response that the ocular lymphoma microenvironment appears to produce.

## Abbreviations

APC: Allophycocyanin; CpG: Cytosine phosphodiester guanine; FITC: Fluorescein isothiocyanate; GFP: Green fluorescent protein; H-TdR: Tritiated thymidine; mAb: monoclonal antibody; ODN: Oligodeoxynucleotide; PBS: Phosphate buffered saline; PCL: Primary cerebral lymphoma; PCNSL: Primary central nervous system lymphoma; PE: Phycoerythrin; PI: Propidium iodide; PIE: PBS-injected eye; PIOL: Primary intraocular lymphoma; RPMI: Roswell Park Memorial Institute; SCL: Subcutaneous lymphoma; TLR: Toll-like receptor.

## Competing interests

The authors declare they have no financial conflicts of interest.

## Authors’ contributions

Contribution: RBA, JC, and SD performed the experiments and wrote the paper. LC and HO provided technical assistance; WHF, CSF, MA, and SF contributed to the writing and to the critical reading of the paper; SF conceived and planned the study. All authors read and approved the final manuscript.

## References

[B1] Chang ZL Important aspects of Toll-like receptors, ligands and their signaling pathways Inflamm Res 2010 59 10 791 808 10.1007/s00011-010-0208-2 20593217

[B2] Dunne A Marshall NA Mills KH TLR based therapeutics Curr Opin Pharmacol 2011 11 4 404 411 10.1016/j.coph.2011.03.004 21501972

[B3] Krieg AM Toll-like receptor 9 (TLR9) agonists in the treatment of cancer Oncogene 2008 27 2 161 167 10.1038/sj.onc.1210911 18176597

[B4] Weiner GJ Liu HM Wooldridge JE Dahle CE Krieg AM Immunostimulatory oligodeoxynucleotides containing the CpG motif are effective as immune adjuvants in tumor antigen immunization Proc Natl Acad Sci USA 1997 94 20 10833 10837 10.1073/pnas.94.20.10833 9380720 PMC23500

[B5] Verthelyi D Ishii KJ Gursel M Takeshita F Klinman DM Human peripheral blood cells differentially recognize and respond to two distinct CPG motifs J Immunol 2001 166 4 2372 2377 11160295 10.4049/jimmunol.166.4.2372

[B6] Hartmann G Krieg AM Mechanism and function of a newly identified CpG DNA motif in human primary B cells J Immunol 2000 164 2 944 953 10623843 10.4049/jimmunol.164.2.944

[B7] Krieg AM Yi AK Matson S Waldschmidt TJ Bishop GA Teasdale R Koretzky GA Klinman DM CpG motifs in bacterial DNA trigger direct B-cell activation Nature 1995 374 6522 546 549 10.1038/374546a0 7700380

[B8] Kuo CC Liang CM Lai CY Liang SM Involvement of heat shock protein (Hsp) 90 beta but not Hsp90 alpha in antiapoptotic effect of CpG-B oligodeoxynucleotide J Immunol 2007 178 10 6100 6108 17475835 10.4049/jimmunol.178.10.6100

[B9] Jahrsdorfer B Muhlenhoff L Blackwell SE Wagner M Poeck H Hartmann E Jox R Giese T Emmerich B Endres S B-cell lymphomas differ in their responsiveness to CpG oligodeoxynucleotides Clin Cancer Res 2005 11 4 1490 1499 10.1158/1078-0432.CCR-04-1890 15746051

[B10] Liang X Moseman EA Farrar MA Bachanova V Weisdorf DJ Blazar BR Chen W Toll-like receptor 9 signaling by CpG-B oligodeoxynucleotides induces an apoptotic pathway in human chronic lymphocytic leukemia B cells Blood 2010 115 24 5041 5052 10.1182/blood-2009-03-213363 20339095 PMC2890142

[B11] Jahrsdorfer B Jox R Muhlenhoff L Tschoep K Krug A Rothenfusser S Meinhardt G Emmerich B Endres S Hartmann G Modulation of malignant B cell activation and apoptosis by bcl-2 antisense ODN and immunostimulatory CpG ODN J Leukoc Biol 2002 72 1 83 92 12101266

[B12] Rubenstein J Ferreri AJ Pittaluga S Primary lymphoma of the central nervous system: epidemiology, pathology and current approaches to diagnosis, prognosis and treatment Leuk Lymphoma 2008 49 Suppl 1 43 51 18821432 10.1080/10428190802311441 PMC4110179

[B13] Donnou S Galand C Touitou V Sautes-Fridman C Fabry Z Fisson S Murine models of B-cell lymphomas: promising tools for designing cancer therapies Adv Hematol 2012 2012 701 704 10.1155/2012/701704 PMC3287022 22400032

[B14] Houot R Levy R T-cell modulation combined with intratumoral CpG cures lymphoma in a mouse model without the need for chemotherapy Blood 2009 113 15 3546 3552 10.1182/blood-2008-07-170274 18941113 PMC2668854

[B15] Weiner GJ The immunobiology and clinical potential of immunostimulatory CpG oligodeoxynucleotides J Leukoc Biol 2000 68 4 455 463 11037965

[B16] Li J Song W Czerwinski DK Varghese B Uematsu S Akira S Krieg AM Levy R Lymphoma immunotherapy with CpG oligodeoxynucleotides requires TLR9 either in the host or in the tumor itself J Immunol 2007 179 4 2493 2500 17675511 10.4049/jimmunol.179.4.2493

[B17] Jahrsdorfer B Weiner GJ CpG oligodeoxynucleotides as immunotherapy in cancer Update Cancer Ther 2008 3 1 27 32 10.1016/j.uct.2007.11.003 19255607 PMC2390897

[B18] Weiner GJ CpG oligodeoxynucleotide-based therapy of lymphoid malignancies Adv Drug Deliv Rev 2009 61 3 263 267 10.1016/j.addr.2008.12.006 19168102

[B19] Galea I Bechmann I Perry VH What is immune privilege (not)? Trends Immunol 2007 28 1 12 18 10.1016/j.it.2006.11.004 17129764

